# Machine Learning-Based Accurate Full-Sib Family Assignment in Sturgeon Using Whole-Genome Sequencing Data

**DOI:** 10.3390/ijms27104359

**Published:** 2026-05-14

**Authors:** Jiayu Yan, Huijuan Li, Tian Dong, Wei Wang, Xiaoyu Yan, Song Bai, Hailiang Song, Hongxia Hu

**Affiliations:** 1 Beijing Key Laboratory of Fisheries Biotechnology, Fisheries Science Institute, Beijing Academy of Agricultural and Forestry Sciences, Beijing 100068, China; jiayuyan164@163.com (J.Y.); lihuijuan@baafs.net.cn (H.L.); yinghua328@163.com (T.D.); raywang8848@163.com (W.W.); yanxiaoyu21@126.com (X.Y.); baisong@baafs.net.cn (S.B.); 2 Key Laboratory of Sturgeon Genetics and Breeding, Ministry of Agriculture and Rural Affairs, Hangzhou 311799, China

**Keywords:** full-sib family assignment, machine learning, whole-genome resequencing, aquaculture breeding, sturgeon

## Abstract

Accurate pedigree reconstruction is essential for genetic evaluation, inbreeding control, and family management in aquaculture breeding programs. In sturgeon, extremely high fecundity and communal rearing during early developmental stages often lead to the loss of family information, making reliable full-sib family assignment a critical challenge. In this study, we developed a machine learning-based framework for full-sib family assignment using simulated genotype datasets and whole-genome resequencing data from Russian sturgeon. Simulation analyses across five machine learning algorithms showed that training set size and marker density were the primary determinants of assignment accuracy. When at least 10 individuals per family were included in the training set, mean identification accuracy exceeded 99% across all evaluated scenarios, and exceeded 99.9% for all methods except XGBoost. In contrast, performance declined when the marker number was reduced to 200. At moderate marker densities (500–1000 markers), performance remained stable, with mean identification accuracy around 99% even when only 3–4 individuals per family were included in the training set. Validation using whole-genome resequencing data (sequencing depth ranging from 9.43× to 11.86×) from 582 individuals representing 19 full-sib families of Russian sturgeon confirmed the simulation findings, with several algorithms achieving assignment accuracies exceeding 99%. These results demonstrate that machine learning provides an accurate and robust approach for full-sib family assignment using genome-wide single nucleotide polymorphism (SNP) data. The proposed framework offers an effective solution for pedigree reconstruction and family identification in sturgeon breeding populations lacking reliable pedigree records.

## 1. Introduction

Sturgeon are economically important aquaculture species due to their high-value products, including caviar and meat, and have become key targets of selective breeding programs in many countries. However, sturgeon broodstock exhibit extremely high fecundity, and offspring from different parental crosses are typically reared communally during early developmental stages, resulting in extensive family mixing. Under such production conditions, it is difficult to maintain accurate pedigree records using traditional tagging or physical separation methods [1,2,3]. Accurate pedigree and family information are essential for effective breeding management, as they provide the basis for genetic evaluation, inbreeding control, balanced family contributions, and the maintenance of genetic diversity within breeding populations [4,5]. The absence of reliable family information may lead to unintended inbreeding accumulation, reduced effective population size, and the gradual loss of favorable genetic variation, ultimately compromising the long-term efficiency and sustainability of breeding programs [6]. Therefore, reliable and scalable methods for full-sib family assignment are critically needed to support genomic pedigree reconstruction and improve breeding management in sturgeon populations.

Breed identification is a relatively well-established task in aquaculture genetics and breeding, playing an important role in genetic resource conservation, stock management, and the maintenance of strain integrity [7,8]. In aquaculture systems, distinct breeds, strains, or populations often exhibit measurable genetic differentiation as a result of geographic isolation, domestication history, or artificial selection [9,10,11]. Leveraging this differentiation, molecular marker-based approaches, particularly those using single nucleotide polymorphisms (SNPs), have been widely applied for breed identification in aquatic species. SNP-based genetic differentiation indices (e.g., Fst), clustering analyses, and principal component analysis (PCA) have demonstrated strong performance in discriminating among aquaculture breeds or populations [12,13,14]. With the increasing availability of genome-wide SNP data, supervised and unsupervised machine learning methods have further improved breed identification accuracy by effectively capturing population structure in high-dimensional genetic datasets [15,16,17].

Compared to breed identification, full-sib family assignment represents a substantially finer-scale and more challenging task in aquaculture breeding systems. Individuals from different families are typically separated by only one or a few generations, resulting in extremely weak genetic differentiation that may be strongly confounded by the overall population structure and within-population genetic diversity [5,18]. Consequently, population differentiation indices commonly used for breed or population identification, such as Wright’s Fst [19] and Delta [20], are not readily applicable to family-level assignment. Traditional marker-based approaches, including microsatellite analyses, may also suffer from elevated error rates and increased genotyping costs when applied to large-scale breeding populations. However, these methods often depend on population-specific marker selection and prior assumptions, which may limit their generalizability across breeding populations. Several software packages, such as COLONY or CERVUS, employ likelihood-based or Bayesian frameworks to perform parentage identification using subsets of highly informative SNPs or microsatellite markers [21,22]. In fish [23,24], including sturgeons, these tools have been widely used to infer the number of contributing families or candidate parents within juvenile cohorts, thereby providing insights into family structure, genetic diversity, and population management [25,26,27,28]. However, these approaches are primarily designed for pedigree reconstruction and parentage inference, rather than for assigning an unknown individual to a specific full-sib family under breeding conditions. This distinction is particularly important in aquaculture, where mixed-family rearing, mass spawning, and incomplete pedigree records often increase the need for accurate individual-level family assignment. Previous studies have shown that the performance of COLONY and CERVUS may be limited in such settings. For example, one recent study reported that COLONY assigned parents to only 62.3% of 1098 individuals [25], while another study found that COLONY did not provide sufficient assignment accuracy for the study objective and that CERVUS identified maternal origin for only 147 of 675 individuals [26]. In this context, machine learning methods offer a promising complementary strategy for genomic family assignment. By modeling complex genomic patterns across genome-wide SNP data, machine learning methods may better capture subtle genetic differences among closely related families and may be more robust to complex genetic backgrounds [29]. In addition, unlike approaches that rely on predefined marker panels, machine learning models can leverage genome-wide SNP information, providing a potentially more generalizable and scalable framework for pedigree inference. Once trained, these models can also be applied to large datasets with high computational efficiency [26]. Despite their increasing use in SNP-based breed identification, systematic investigations of machine learning approaches for full-sib family assignment in aquaculture remain limited. Furthermore, the extent to which population genetic structure influences family assignment accuracy has not been comprehensively evaluated.

To address these challenges, we first used simulated family populations with controlled genetic backgrounds to evaluate the performance of machine learning methods under different conditions, including the number of families, training set size, SNP density, and parental composition. We then validated the simulation-based findings using real genome-wide SNP data from sturgeon to further assess their robustness and practical applicability in aquaculture breeding systems. This study establishes a scalable machine learning framework for full-sib family assignment in aquaculture and identifies key factors affecting model performance.

## 2. Results

### 2.1. Impact of Training Set Size on Accuracy

**Figure 1:**
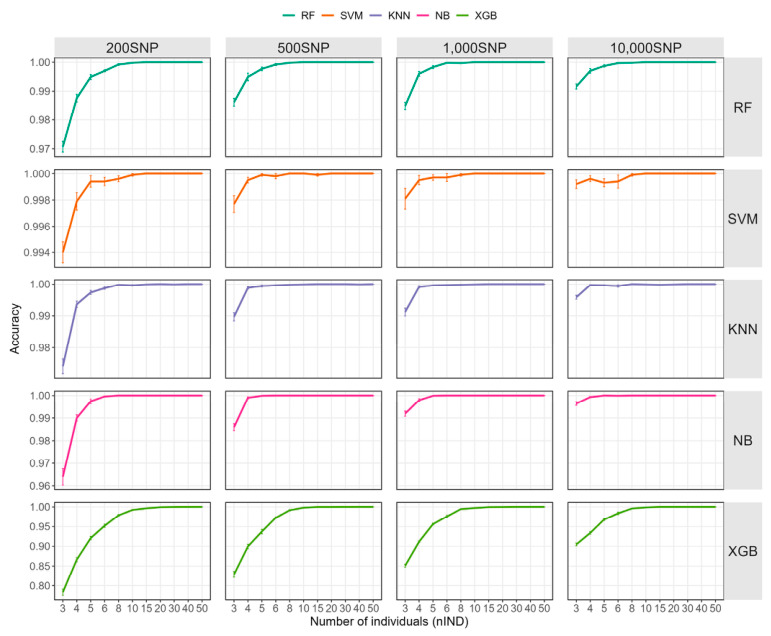
Impact of training set size on accuracy. RF: random forest; KNN: K-nearest neighbors; XGB: extreme gradient boosting; NB: naive bayes; SVM: support vector machine.

The impact of different numbers of individuals in the training set on identification accuracy is shown in Figure 1 and Supplementary Table S1. Overall, the accuracy of all machine learning methods increased rapidly with the expansion of the training set size, reaching a mean accuracy of more than 99% across all models and SNP densities at *nIND* (number of individuals per family) = 10. When the number of individuals exceeded 20 per family, the accuracy of all machine learning models across various *nSNP* (number of SNPs used in the model) levels exceeded 99.9%. In the comparison of different algorithms, Support vector machine (SVM) consistently achieved the highest average identification accuracy, with mean accuracy exceeding 99% under all training set sizes and SNP densities, although slight fluctuations were observed at larger training set sizes. In contrast, Extreme gradient boosting (XGBoost) displayed the lowest average accuracy. For example, with 1000 SNPs, its accuracy decreased to 95.61% ± 0.79% at *nIND* = 5 and further dropped to 85.09% ± 1.23% at *nIND* = 3. The number of SNPs also influenced identification performance. As SNP density decreased, overall accuracy declined. When 1,000 SNPs were used, reducing *nIND* from 10 to 3 caused only modest declines in naive Bayes (NB), SVM, K-nearest neighbors (KNN), and Random forest (RF), with their accuracies decreasing from more than 99.99% to approximately 99%; in contrast, XGBoost declined to 85.09% ± 1.23%. When the number of SNPs was further reduced to 200, the effect became much more pronounced: as *nIND* decreased from 10 to 3, the accuracies of NB, SVM, KNN, and RF declined from more than 99.96% to as low as 96.40% ± 1.15%, whereas XGBoost showed a substantially larger decrease to 78.36% ± 2.44%. Regarding identification stability, when *nIND* > 10, NB and RF demonstrated stable and perfect identification across all replicates (100.00% ± 0.00%), whereas KNN and SVM did not reach 100% accuracy in a few scenarios, with accuracies of 99.99% ± 0.03%.

### 2.2. Impact of Hyperparameter Optimization on Accuracy

**Figure 2:**
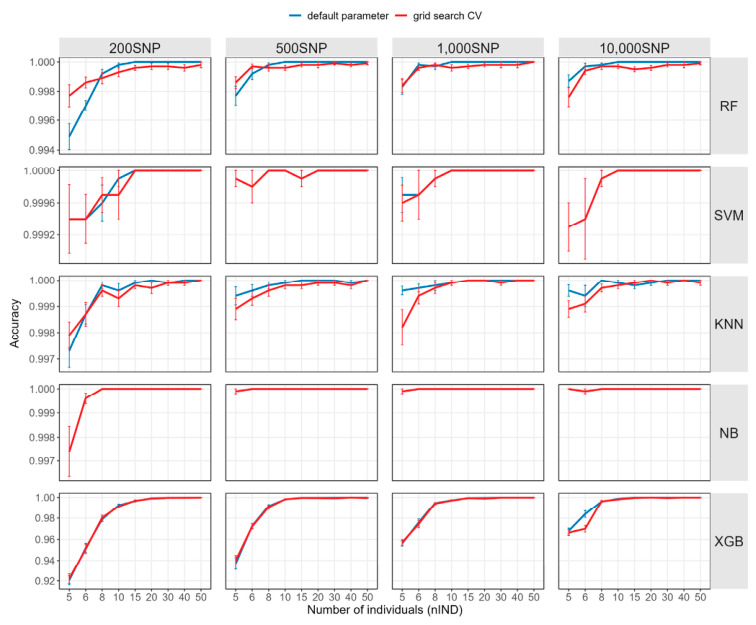
Impact of hyperparameter optimization on accuracy. RF: random forest; KNN: K-nearest neighbors; XGB: extreme gradient boosting; NB: naive bayes; SVM: support vector machine.

We further employed GridSearchCV to perform hyperparameter optimization for each machine learning method to evaluate the impact of hyperparameter adjustment on parentage identification accuracy under different *nSNP* levels and training set sizes. Since hyperparameter optimization utilized five-fold cross-validation, groups with fewer than 5 individuals per family in the training set were excluded from this part of the analysis. The identification accuracy after hyperparameter optimization under different *nSNP* levels is shown in Figure 2 and Supplementary Table S2. Overall, the improvement in final classification accuracy provided by hyperparameter optimization was very limited. In most methods, the optimized accuracy was close to the results obtained with default parameters, and the largest improvement observed was only 0.28 percentage points, from 99.49% ± 0.28% to 99.77% ± 0.24%, in RF with 200 SNPs and *nIND* = 5. In some model and data-size settings, accuracy after hyperparameter optimization was even slightly lower than that obtained with the default parameters, particularly for RF when *nIND* > 8 and in most KNN settings. This phenomenon may be related to the small genetic differences between families, the fact that models were already near-optimal with default parameters, or the randomness introduced by the cross-validation process.

### 2.3. Impact of Sire Number on Accuracy

**Figure 3:**
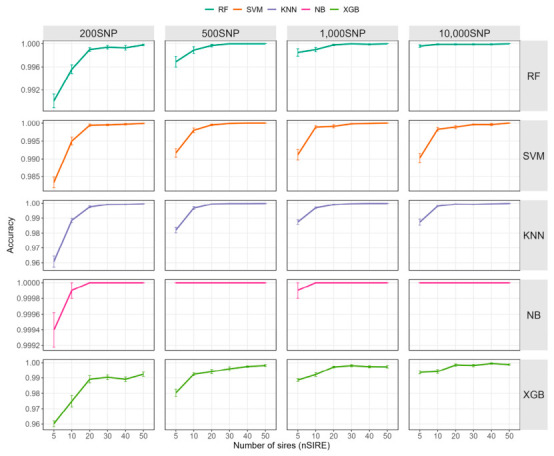
Impact of sire number on accuracy. RF: random forest; KNN: K-nearest neighbors; XGB: extreme gradient boosting; NB: naive bayes; SVM: support vector machine.

Given that the results from the previous section indicated limited improvement in classification performance via hyperparameter optimization, we continued to use default parameters for model training in the subsequent analysis. In this section, we fixed the training set size at *nIND* = 10—a size shown to achieve more than 99% identification precision in all model and SNP densities—and evaluated the impact of different sire numbers (*nSIRE*) on identification accuracy (Figure 3 and Supplementary Table S3). Overall, as the number of sires retained per generation decreased, the accuracy of all models showed a declining trend, with XGBoost exhibiting relatively lower overall accuracy. When *nSIRE* < 20, the accuracy of all models dropped noticeably.

Differences in robustness were observed among different algorithms. NB was the least sensitive to changes in sire number, achieving 100.00% identification accuracy across different *nSIRE* levels at almost all SNP densities; only when *nSIRE* = 5 and *nSNP* = 200 did its accuracy decrease slightly to 99.94% ± 0.07%. RF also demonstrated good stability, with its accuracy declining from 100.00% to a minimum of 99.01% ± 0.38% across the tested scenarios. In contrast, KNN, SVM, and XGBoost showed larger declines as both *nSIRE* and *nSNP* decreased. Specifically, KNN declined from 99.99% ± 0.03% to 96.08% ± 1.18%, SVM from 100.00% to 98.33% ± 0.48%, and XGBoost from 99.95% ± 0.16% to 96.02% ± 0.58%, indicating that XGBoost was the most sensitive to reductions in sire number, particularly under lower SNP densities. The effect of SNP number was also clear: when *nSNP* ≥ 500, the differences in accuracy among different *nSIRE* conditions were relatively small, whereas at *nSNP* = 200, the variation among *nSIRE* conditions became much larger.

### 2.4. Impact of Dam (Family) Number on Accuracy

**Figure 4:**
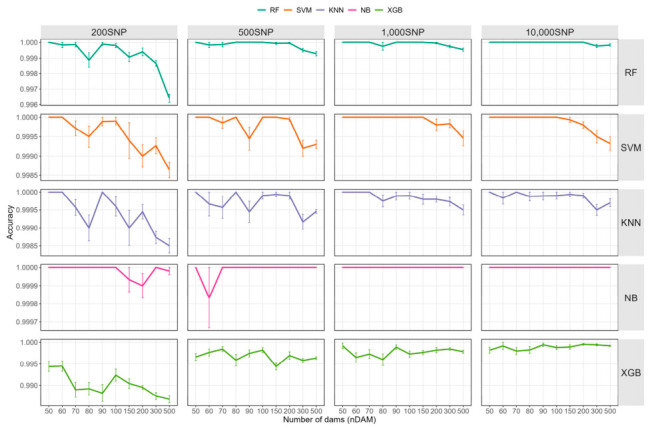
Impact of dam number on accuracy. RF: random forest; KNN: K-nearest neighbors; XGB: extreme gradient boosting; NB: naive bayes; SVM: support vector machine.

As the number of families (*nDAM*) increased, the identification accuracy of all algorithms generally showed a trend of slight fluctuation and a marginal decline (Figure 4 and Supplementary Table S4). This trend remained relatively stable when more than 500 SNPs were used, with all models maintaining identification accuracies above 99%. In contrast, under the 200SNP condition, both the decline in accuracy and the magnitude of fluctuation became more pronounced; for XGBoost, accuracy fell below 99% when the number of families was relatively large, reaching a minimum of 98.68% ± 0.27%. These results indicate that as the number of families increases and the complexity of the identification task rises, the impact of low SNP density on model performance becomes particularly prominent, while appropriately increasing the number of SNPs helps achieve more stable classification results in multi-family scenarios.

### 2.5. Training Time Costs

**Figure 5:**
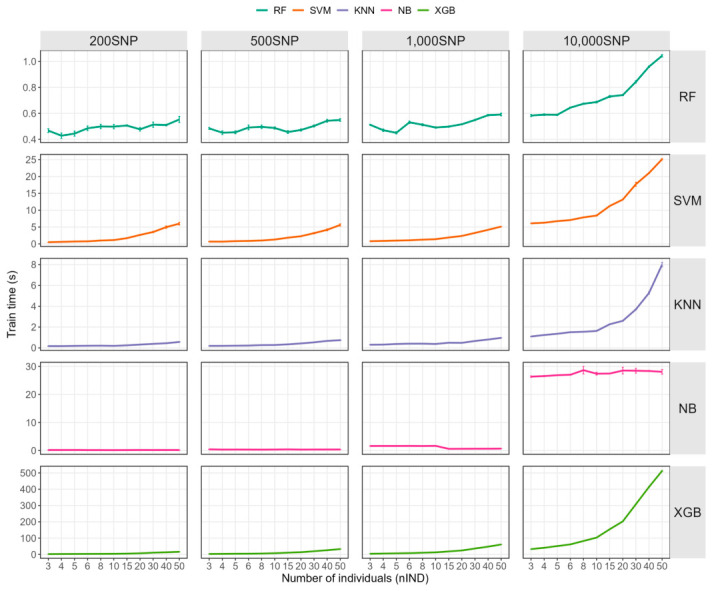
Training time (s) under different individual sizes, machine-learning method and SNP scales. RF: random forest; KNN: K-nearest neighbors; XGB: extreme gradient boosting; NB: naive bayes; SVM: support vector machine.

Although model identification accuracy improved with increasing data volume, the computational time required for training also increased. We quantified the time consumption during the training process for different training set individual sizes and different machine learning methods (Figure 5 and Supplementary Table S5). Training time varied substantially among algorithms. Overall, RF and KNN were the fastest methods, with RF completing training in less than 1 s across all tested conditions, whereas XGBoost was the most time-consuming method, with the longest training time reaching 512.4310 ± 9.0959 s. Among the different algorithms, the training time of GaussianNB was least affected by the training set size, with its computational cost primarily determined by the *nSNP*. In contrast, the other machine learning methods (including KNN, SVM, RF, and XGBoost) all showed varying degrees of extended training time as the size of individuals in the training set increased, although the impact of *nSNP* on time consumption remained the most significant factor overall.

### 2.6. Validation Using Whole-Genome Resequencing Data of Sturgeon

**Figure 6:**
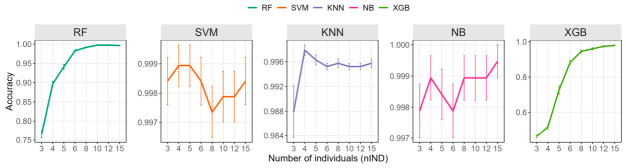
Full-sib family assignment accuracy across training set sizes in sturgeon based on whole-genome resequencing data. RF: random forest; KNN: K-nearest neighbors; XGB: extreme gradient boosting; NB: naive bayes; SVM: support vector machine.

Whole-genome sequencing of 582 sturgeon individuals generated high-quality genomic data with an overall alignment rate of 92.19%, resulting in a mean sequencing depth of 11.37× per individual (ranging from 9.43× to 11.86×). Variant calling identified a total of 41,282,218 SNPs, of which 197,679 high-quality SNPs were retained after stringent quality filtering for downstream machine learning analyses. The impact of different training set sizes on family assignment accuracy for the 19 sturgeons full-sib families is shown in Figure 6 and Supplementary Table S6. Consistent with the observations from simulated datasets, increasing the number of individuals per family improved family assignment accuracy. NB, KNN, and SVM all achieved accuracies above 99% across all *nIND* levels, with one exception for KNN, which yielded a mean accuracy of 98.79% ± 1.34% when only 3 individuals per family were used for training. In addition, except for XGBoost, all methods exceeded 99% accuracy when the number of individuals per family reached 10, whereas XGBoost achieved 96.00% ± 2.00% at *nIND* = 10. RF and XGBoost showed larger declines in accuracy as the training set size decreased; when *nIND* was reduced to 3, their accuracies dropped to 76.53% ± 2.93% and 46.16% ± 2.48%, respectively. It is noteworthy that, compared to simulated data, the real genotype dataset showed slightly lower identification accuracy at the same training set sizes especially for RF and XGBoost methods, indicating that biological variation and potential genotyping noise in real data can limit model performance. Despite this, the overall trend remained consistent: increasing the *nIND* improved prediction accuracy.

## 3. Discussion

In sturgeon breeding programs, accurate pedigree and family information is essential for genetic evaluation, inbreeding control, and the maintenance of genetic diversity. However, due to the extremely high fecundity of broodstock and the common practice of communal rearing during early developmental stages, family information is often lost or incomplete in practical aquaculture production. Molecular marker-based parentage and family assignment therefore provides an effective approach for reconstructing pedigree relationships in sturgeon populations. Over the past two decades, research on parentage identification based on SNP data has achieved high accuracy in various animal species. Previous studies demonstrated that reliable pedigree inference could be achieved using a limited number of highly informative SNP loci [24,30]. However, most of these studies adopted likelihood or Bayesian inference frameworks developed based on earlier microsatellite markers. Their core methodology still relies on selecting a small number of highly informative markers and is highly sensitive to genotyping errors and allele frequency estimation.

With the continuous decline in whole-genome resequencing costs and the widespread application of SNP arrays in aquatic species, the scale and dimension of genotyping data have significantly increased, providing possibilities for testing new analytical frameworks. Compared with traditional probability model-based methods, machine learning methods do not require prior assumptions about genetic models or allele frequency distributions. They can directly use high-dimensional SNP genotypes as input to automatically learn the overall genetic patterns between different families from the data. ML models can, in principle, capture non-linear interactions, which may contribute to generalization and robustness. This “data-driven” analytical approach provides a feasible path, provides an alternative approach that may be suitable when genetic differentiation between families is low, for conducting pedigree identification in scenarios with weak genetic differentiation between families.

### 3.1. Impact of Training Set Individual Size on Accuracy

Under actual breeding and production conditions, the number of samples available for establishing a training set is often limited by cost and operational constraints. Therefore, minimizing the number of training samples while ensuring identification accuracy holds important practical significance. However, an excessively small sample size may fail to fully characterize genetic variation within families, thereby affecting model stability and generalization. In similar machine learning breed identification studies, it has been noted that when the number of individuals used for training per population reaches approximately 10, models can achieve near 99% identification accuracy [17,31,32].

The results of this study indicate that training set size is one of the most critical factors affecting parentage identification accuracy. When the number of individuals per family used for training reached 20, all machine learning methods achieved mean identification accuracies above 99.9% across different SNP density conditions. However, when the number of training individuals was small (e.g., 5–10 per family), performance differences among algorithms become apparent. Within this sample size range, XGBoost showed the largest decrease in performance, with mean accuracy dropping to 92.06% ± 1.34% under the condition of 5 training individuals per family and 200 SNPs, whereas the other methods maintained mean accuracy above 99%.

When the number of training individuals per family was further reduced (e.g., 3–4), SVM still achieved overall mean accuracies above 99%. KNN and NB showed comparable performance, while RF yielded lower mean accuracy than both KNN and NB under these conditions. In addition, NB demonstrated the highest robustness: when the number of individuals per family exceeded 8, it correctly classified all validation samples across all experimental settings. In contrast, SVM and KNN still exhibited occasional misclassifications even when the number of training individuals per family was relatively large.

These results suggest that in the high-similarity classification task of parentage identification, adequate training samples are irreplaceable for accurately capturing weak genetic differences between families, while the robustness of different machine learning methods varies significantly under low sample conditions. Insufficient training individuals reduce the ability to resolve weak genetic differences, thereby compromising the reliability of downstream kinship management and inbreeding monitoring.

### 3.2. Impact of SNP Number on Identification Performance

In parentage identification, the number of SNPs adopted is essentially a trade-off made by researchers between the risks of false negative and false positive identification [33]. Previous SNP panel-based studies have shown that the range of SNP numbers capable of reliable identification is broad, ranging from dozens [34] to thousands of loci [33]. In this context, recent studies employing machine learning for family or population identification have generally selected SNP data within this range. Furthermore, some studies point out that characteristic SNPs selected based on genetic differentiation indices may yield different identification accuracy compared to randomly selected SNPs [31].

In this study, we did not pre-select characteristic SNPs but aimed to construct a machine learning parentage identification framework with stronger generalization capabilities. Therefore, we constructed independent datasets for different SNP density levels to systematically evaluate the impact of SNP number itself on identification performance. The results showed that the number of SNP decreased, mean identification accuracy declined across all algorithms. In addition, lower SNP densities amplified the negative effects of reduced training sample size or fewer sires on accuracy, a pattern that was most evident when only 200 SNPs were used.

In contrast, when the number of SNPs reached 500 or more, mean accuracy still showed a slight decrease as SNP number declined, but the magnitude of this decrease was relatively small. This indicates that when SNP density exceeds 500 loci, further increases in SNP number result in only limited improvements in identification accuracy.

Combined with the analysis of training time costs, it is evident that under the 10,000 SNP conditions, model training time increased significantly, whereas under 500–1,000 SNP conditions, training efficiency was higher and reducing SNP number from 10,000 to 500 resulted in a decrease in mean identification accuracy of less than 1%. Therefore, in actual production applications, adopting medium-scale SNP data may be more conducive to balancing computational efficiency and identification performance. In practical production settings, using a moderate-scale SNP dataset may better balance computational efficiency and assignment performance, and can serve as a management threshold that jointly considers accuracy and cost under resource-limited conditions.

### 3.3. Impact of Family Genetic Structure on Accuracy

Besides training set size and SNP number, the internal genetic structure of families is also an important factor affecting identification performance. This study systematically evaluated the impact of changes in the number of sires and dams (families) on parentage identification accuracy. Results indicated that reducing the number of sires had the largest impact on accuracy: when the number of sires dropped to 5, mean identification accuracy decreased across all machine learning methods under low SNP density conditions; however, when the SNP number reached 500 or more, overall identification performance remained at a high level. Therefore, in scenarios with a low number of sires, marker density and/or training sample size should be increased to reduce potential bias in family assignment.

This result forms a meaningful contrast with previous studies based on parentage assignment. For example, Sherman et al. based on microsatellite markers and exclusion methods, pointed out that as the number of candidate fathers increases, the difficulty of parentage assignment rises significantly because the number of potential fathers to be excluded increases [35]. Unlike that study, the present study employs a family classification framework based on overall genotype patterns. In this context, a reduction in the number of sires lowers the degree of genetic differentiation between different families, making the distribution of offspring from different sires closer in genotype space, thereby increasing the difficulty for the classification model to distinguish families.

In contrast, an increase in the number of dams (families) only caused slight fluctuations in identification accuracy, with no obvious systematic downward trend observed. This suggests that within the examined parameter range, machine learning methods have strong adaptability to increases in the number of families. However, to ensure identification robustness under complex family structures, it is still necessary to maintain at least a moderate number of SNPs to compensate for the information loss caused by reduced genetic differentiation.

### 3.4. Impact of Hyperparameter Optimization on Identification Performance

Hyperparameter tuning is usually considered an important means to improve machine learning model performance. In this study, we further evaluated the practical effectiveness of a GridSearchCV-based hyperparameter optimization strategy in the parentage identification task. Overall results showed that under different SNP numbers and training set sizes, the improvement in final identification accuracy provided by hyperparameter optimization was generally limited. Most models showed no significant performance difference between default and optimized parameters, and in some cases, there was even a slight decline.

This phenomenon is to some extent expected. First, in the task of parentage identification, the genetic differences between different families are inherently weak. When the SNP number reaches a medium level, model performance is more likely constrained by the upper limit of genetic information rather than algorithm parameter settings. Previous studies have noted that when input feature information is sufficient and the signal-to-noise ratio is high, some classic machine learning models can achieve near-optimal classification performance with default or empirical parameters, and further fine-tuning often brings limited gains [36]. Second, hyperparameter optimization usually relies on cross-validation to estimate the generalization performance of the model. In scenarios with a limited number of families and small sample sizes per family, the cross-validation partition itself may introduce additional random fluctuations, thereby affecting the stability of optimized parameters on an independent test set.

In summary, this study suggests that in the high-similarity classification problem of parentage identification, rather than relying on complex hyperparameter searches, priority should be given to key data-level factors such as SNP number, training set size, and family genetic structure.

### 3.5. Validation on Real Sturgeon Data

To assess the applicability of the machine learning framework for full-sib family assignment developed based on simulated data, we applied it to whole-genome resequencing data from 19 Russian sturgeon full-sib families. The results on real data demonstrated that all algorithms were able to assign individuals to families with high stability. NB, KNN, and SVM consistently achieved identification accuracies exceeding 99% across all training set sizes, with one exception for KNN, which yielded a mean accuracy of 98.79% ± 1.34% when only 3 individuals per family were used for training. In contrast, RF and XGBoost showed reduced accuracy when the number of training individuals was small. However, when the training set included more than 10 individuals per family, RF achieved mean accuracies above 99%, whereas XGBoost showed slightly lower performance, with a mean accuracy of 96.00% ± 2.00%.

In this study, the data used, particularly the real whole-genome resequencing data used for validation, is characterized by a feature count (SNP count) that is much larger than the sample size (the number of individuals per family). Especially when the number of individuals per family (*nIND*) decreases to 5 or fewer, the ratio of features to samples increases drastically. This scenario is referred to as the high-dimensional low sample size (HDLSS) problem in machine learning, which presents higher challenges for certain classifiers [37,38]. For tree-based algorithms such as RF and XGBoost, each “tree” becomes increasingly susceptible to noise as the number of features grows. This characteristic makes these algorithms more demanding in terms of the required size of the training sample. Therefore, we believe that it is crucial to reasonably select the appropriate classifier and machine learning method based on the number of features (e.g., the number of SNPs used for family assignment) and sample size (the number of individuals per family used for training) when performing family assignments.

These findings indicate that building on the reasonable selection of machine learning methods and data size, the framework performs robustly not only on simulated datasets but also on actual aquaculture genotype data, confirming its practical applicability. The slightly lower accuracy observed in real data compared with simulated scenarios is expected, as real aquaculture populations often contain additional sources of variation, including uneven parental contributions, historical relatedness among broodstock, and environmental influences on offspring performance, all of which may reduce the genetic distinctiveness among families. In real-world breeding management, this suggests that accurate family assignment and tracking can be achieved using a relatively limited number of training samples and a moderate-scale SNP dataset, providing reliable support for pedigree maintenance, breeding strategy implementation, and inbreeding control in aquaculture populations.

### 3.6. Limitations and Future Directions

Although the machine learning framework proposed in this study demonstrated good robustness in both simulated datasets and real whole-genome resequencing data from Russian sturgeon, and showed promising generalizability through repeated validation using multiple randomized datasets, several limitations should still be acknowledged. First, validation on real data was limited to a single species (Russian sturgeon) and 19 full-sib families, representing a relatively restricted sample scope. Further evaluation across additional aquaculture species, breeding populations, and populations with different genetic backgrounds will be necessary to comprehensively assess the generalizability of the framework. Second, the present study focused primarily on full-sib family assignment within the same generation, whereas more complex pedigree structures may occur in practical breeding systems, such as half-sib relationships, overlapping generations, or the continuous introduction of new families. The applicability of the current framework under such scenarios therefore remains to be further explored. Finally, although genome-wide SNP data already achieved satisfactory assignment accuracy in the present study, further optimization is still needed to identify smaller and more cost-effective SNP panels with comparable discriminatory power.

By addressing these limitations, the practical utility of this framework in aquaculture breeding could be further improved. In addition to providing technical support for pedigree identification, inbreeding control, and the maintenance of genetic diversity in a wider range of species, the framework may also contribute to breeding management practices such as family tracking, parental combination evaluation, optimization of broodstock selection, and the long-term genetic improvement of breeding populations.

## 4. Material and Methods

### 4.1. Simulation Data

Simulation genotype datasets were generated using QMSim 2.0 [39]. For each independent simulation replicate, the random seed was generated and recorded by QMSim to ensure reproducibility of the simulated datasets. For each independent simulation replicate, a historical population (hp) was first constructed to establish linkage disequilibrium and genetic diversity. The historical population was simulated for 1,000 non-overlapping generations, with each generation consisting of 600 males and 600 females. During the historical period, matings were random, and no selection or migration was applied.

### 4.2. Historical Population and Family Generation

From the last generation of the historical population, a predefined number of males (*nSIRE*) and females (*nDAM*) were randomly sampled to form the founder population. The founders were randomly mated to generate the F1 generation, with each full-sib family producing 100 offspring individuals. In each subsequent generation, equal numbers of males and females were randomly selected as breeders to maintain a constant family structure. This mating scheme was repeated across generations until the F6 generation was reached, resulting in *nDAM* full-sib families in the F6 population, each family comprising 100 individuals with simulated genotype data used for downstream analyses.

### 4.3. Marker Genotype Simulation

To generate marker genotypes, all loci were initialized as monomorphic in the base population, and polymorphism was introduced through mutation in subsequent generations. Genetic drift acting over generations allowed the population to reach mutation-drift equilibrium. In this study, a total of 10,000 SNP markers were simulated to mimic a commonly used low-density SNP chip in aquaculture breeding. Markers were assumed to be evenly distributed across the genome. The mutation rate for SNP markers was set to 2.5 × 10^−5^. The simulated genotypes were used as the basis for all subsequent full-sib family identification analyses.

### 4.4. Experimental Scenarios and Parameter Settings

Multiple simulation scenarios were constructed to assess the effects of different factors on family assignment accuracy.

(1) Effect of training sample size. To evaluate the impact of training sample size, the numbers of sires and dams were fixed at *nSIRE* = 50 and *nDAM* = 100. For each family, 10 individuals were first randomly selected as the validation set, and then *nIND* = 3, 4, 5, 6, 8, 10, 15, 20, 25, 30, 40, or 50 individuals were sampled per family from the remaining individuals as the training set. This sampling procedure was performed using the fixed random seed specified at the beginning of the program to ensure reproducibility while preserving randomness.

(2) Effect of parental composition. Because changes in parental composition alter the genetic structure among families, independent simulation datasets were generated for each scenario. The training sample size was fixed at *nIND* = 10 individuals per family. To assess the effect of sire number, the number of sires (*nSIRE*) was set to 5, 10, 20, 30, 40, or 50, with the number of dams fixed at 100. To assess the effect of dam number, the number of dams (*nDAM*) was set to 50, 60, 70, 80, 90, 100, 150, 200, 300, or 500.

(3) For each of the above parameter combinations, genotype datasets were generated under different SNP density levels, including 10,000 SNPs (representing a commonly used 10K SNP panel), as well as 1,000, 500, and 200 SNPs, which are commonly used in machine learning, with simulated SNP marker datasets generated using the methods described above.

### 4.5. Real Data of Sturgeons

Russian sturgeon (*Acipenser gueldenstaedtii*) used in this study were obtained from Hangzhou Qiandaohu Xunlong Sci-tech Co., Ltd. (Hangzhou, China), a commercial aquaculture facility maintaining standardized breeding management and pedigree records. In 2012, artificial fertilization of 251 broodstock individuals (78 females and 173 males) produced 192 full-sib families. From the descendants of these families, a total of 582 individuals representing 19 full-sib families were sampled for the present study. The number of individuals per family was provided in Table 1. Fin tissue samples were collected and preserved in absolute ethanol for subsequent genetic analysis. All fish were reared under uniform aquaculture conditions at the facility, with consistent water quality, temperature, and feeding regimes. The sturgeon used in this study were part of routine aquaculture production for caviar and meat, and no experimental procedures involving live animals were conducted specifically for this research. Therefore, no animal experimental ethics approval was required.

Genomic DNA was extracted from fin tissue using the phenol-chloroform protocol. Whole-genome sequencing of 582 individuals was performed on the DNBSEQ-T7 platform (MGI Tech Co., Ltd., Shenzhen, China) using 150 bp paired-end libraries. Sequencing reads with Phred quality scores above 20 were retained and aligned to the sterlet reference genome (assembly ASM1064508v1) [40] using BWA v0.7.17 [41]. Alignment files in SAM format were converted to BAM using SAMtools v1.2 [42], and PCR duplicates were identified and removed with Picard tool [43]. SNPs were called using the UnifiedGenotyper module in GATK v3.5 [44] with the parameter “--sample-ploidy 2”. This setting was applied because previous studies have indicated that the Russian sturgeon genome exhibits a relatively high degree of functional diploidization [45]. Therefore, genotypes at each locus can be reasonably represented using two alleles, which facilitates downstream genomic analyses. Stringent filtering thresholds were applied to ensure high-confidence variant calls, including FisherStrand < 60, Quality ≥ 50, Quality by Depth ≥ 2.0, and Read Position Rank Sum < −8.0. To mitigate missing genotypes and improve the robustness of genomic predictions, imputation was performed with Beagle v5.5 [46] using default settings. Subsequent quality control in PLINK v1.9 [47] excluded variants with minor allele frequencies less than 0.2. A relatively stringent minor allele frequency (MAF) threshold was applied because the primary goal of this study was to distinguish between closely related full-sib families. Compared with rare variants, SNPs with higher minor allele frequencies are generally more informative for fine-scale family differentiation and are less susceptible to the influence of potential sequencing errors. Furthermore, to reduce redundancy among markers and minimize the potential influence of correlated SNPs on model performance, linkage disequilibrium (LD) pruning was carried out using the --indep-pairwise function in PLINK. Specifically, SNPs were evaluated within sliding windows spanning 50 kb, with a step size of 5 kb between successive windows. Within each window, one SNP from each pair showing a squared correlation coefficient (*r*^2^) greater than 0.2 was removed. A stringent LD threshold (*r*^2^ < 0.2) was used to reduce the overrepresentation of linked genomic regions, as highly correlated SNPs may introduce redundant information and bias downstream machine-learning analyses. After these procedures, a total of 197,679 high-confidence SNPs were retained for downstream analyses.

### 4.6. Machine Learning Methods

We compared five machine learning methods that are commonly used for high-dimensional classification problems and have broad applications in fields such as genomic selection and breed identification. These methods include random forest (RF), support vector machine (SVM), naive bayes (NB), K-nearest neighbors (KNN), and extreme gradient boosting (XGBoost). RF, SVM, NB, and KNN were implemented using scikit-learn [48] in Python 3.11.5, while XGBoost was implemented using the Python xgboost package [49]. For all analyses, the random seed was fixed at 42 at the beginning of each run to ensure reproducibility.

#### 4.6.1. Extreme Gradient Boosting (XGBoost)

XGBoost is an ensemble learning method based on gradient boosting that achieves efficient classification by constructing tree models with error-correction capabilities [49]. The algorithm implements a scalable, end-to-end tree boosting system widely adopted in machine learning competitions and practical applications. The core principle of XGBoost is to build an additive model by iteratively adding new trees that correct the residual errors of the existing ensemble. Given a dataset with n samples and m features, the prediction for sample i at iteration t is:

$${\hat{y}}_{i}^{\left(t\right)}=\sum_{k=1}^{t}f_{k}(x_{i})$$

where $f_{k}$ represents the *k*-th decision tree. The objective function to be minimized at each iteration combines a loss function and a regularization term:

$$L^{\left(t\right)}=\sum_{i=1}^{n}l(y_{i},{\hat{y}}_{i}^{\left(t-1\right)}+f_{t}(x_{i}))+\Omega (f_{t})$$

$$\Omega (f_{t})=\gamma T+\frac{1}{2}\lambda \sum_{j=1}^{T}w_{j}^{2}$$

where $L^{\left(t\right)}$ is the loss function at iteration t, $y_{i}$ is the true label of sample i, ${\hat{y}}_{i}^{\left(t-1\right)}$ is the predicted value of sample i at the previous iteration, $f_{t}(x_{i})$ is the prediction from the t-th decision tree for sample i, $\Omega (f_{t})$ is the regularization term for the t-th tree, T is the number of leaves in tree $f_{t}$, $w_{j}$ is the weight (score) of the j-th leaf in tree $f_{t}$, γ is the regularization parameter that controls the complexity of the tree, λ is the regularization parameter that controls the magnitude of the weights.

#### 4.6.2. Random Forest (RF)

RF is an ensemble model constructed from multiple randomized decision trees [50]. The method combined bagging sampling approach and the random selection of features to construct a collection of decision trees with controlled variation. Classification is completed through majority voting on the prediction results of multiple trees, demonstrating strong generalization capabilities and resistance to overfitting. Formally, a random forest classifier is defined as a collection of tree-structured classifiers:

$$\left\{h(x,\Theta_{k}),|k=1,2,\ldots ,K\right\}$$

where $h(x,\Theta_{k})$ is the prediction from the k-th decision tree for input x, $\Theta_{k}$ represents the random vector controlling the growth of the k-th tree, K is the total number of trees in the ensemble. For classification problems, the final prediction is determined by majority voting:

$$\hat{y}=mode\{h_{1}(x),h_{2}(x),\ldots ,h_{K}(x)\}$$

where $h_{1}(x),h_{2}(x),\ldots ,h_{K}(x)$ are the predictions of the K trees, $\hat{y}$ is the final prediction, determined by the majority vote.

#### 4.6.3. Naive Bayes (NB)

NB is a probabilistic classification method based on Bayes’ theorem, characterized by high computational efficiency and excellent stability [51]. The classifier assumes conditional independence among features given the class label, which greatly simplifies the computation of posterior probabilities. Given an input feature vector x = ($x_{1}$, $x_{2}$, …, $x_{m}$) and class labels $C_{k}$, Bayes’ theorem states: Under the naive independence assumption, the likelihood can be factorized as:

$$P(x|C_{k})=\prod_{j=1}^{m}P(x_{j}|C_{k})$$

The classification rule assigns the sample to the class with the highest posterior probability:

$$\hat{y}=arg\;\underset{C_{k}}{max}P(C_{k})\prod_{j=1}^{m}P(x_{j}|C_{k})$$

where $\hat{y}$ is the predicted class, $P(C_{k})$ is the prior probability of class $C_{k}$, $P(x_{j}|C_{k})$ is the conditional probability of feature $x_{j}$ given the class $C_{k}$, $\arg{max}_{C_{k}}$ denotes the class with the highest posterior probability.

#### 4.6.4. Support Vector Machine (SVM)

SVM achieves classification by constructing hyperplanes that maximize the classification margin and is suitable for high-dimensional data analysis [52,53]. The method conceptually maps input vectors to a high-dimensional feature space where a linear decision surface is constructed with special properties that ensure high generalization ability. For non-linearly separable data, a soft margin is introduced with slack variables $\xi_{i}$:

$$\underset{w,b,\xi }{min}\frac{1}{2}∥w{∥}^{2}+C\sum_{i=1}^{n}\xi_{i}$$

where w is the weight vector (parameters of the hyperplane), b is the bias term, $\xi_{i}$ is the slack variable for sample i (allowing for some misclassification), C is the regularization parameter, controlling the trade-off between margin maximization and classification error, n is the total number of samples. The margin subject to:

$$y_{i}(w^{⊤}x_{i}+b)\geq 1-\xi_{i},\xi_{i}\geq 0$$

where $y_{i}$ is the true class label for sample i, $x_{i}$ is the feature vector for sample i, $w^{⊤}x_{i}+b$ is the decision function for sample i.

As the classifier requires one-hot encoded input, we first used OneHotEncoder() in sklearn to convert the genotypes into one-hot encoding based on the three genotype categories. Given the extremely high feature dimensionality following one-hot processing, we further used PCA() in sklearn to reduce the feature dimensions to 100 to improve model computational efficiency.

#### 4.6.5. K-Nearest Neighbors (KNN)

KNN is a distance-based non-parametric method that classifies samples by comparing the categories of the nearest K neighbors in the training set [54,55]. The method assigns an unclassified sample point the classification of the majority among its K nearest neighbors. Given a test sample x, the algorithm identifies the set $N_{K}(x)$ of K training samples closest to x according to a specified distance metric. The predicted class is determined by majority voting:

$$\hat{y}=arg\;\underset{c}{max}\sum_{i\in N_{K}(x)}I(y_{i}=c)$$

where $\hat{y}$ is the predicted class for the test sample x, $N_{K}(x)$ is the set of K nearest neighbors of the test sample x, $y_{i}$ is the true class label for the i-th neighbor, I(⋅) is the indicator function, which returns 1 if the condition is true and 0 otherwise.

For genotype encoding, we adopted the Hamming distance as the similarity metric instead of the Euclidean distance to make it more suitable for discrete genotype data. The Hamming distance between two genotype vectors $g_{1}$ and $g_{2}$ of length m is defined as:

$$d_{H}(g_{1},g_{2})=\sum_{j=1}^{m}I(g_{1j}\neq g_{2j})$$

where $g_{1}$ and $g_{2}$ are the genotype vectors of length m, $g_{1j}$ and $g_{2j}$ are the genotypes at the j-th position in vectors $g_{1}$ and $g_{2}$, $I(g_{1j}\neq g_{2j})$ is 1 if the genotypes differ at position j and 0 if they are the same.

This metric counts the number of positions at which the corresponding genotypes differ, which is more appropriate for categorical SNP data encoded as 0/1/2 [56]. The encoded SNP matrix was directly used as input for the machine-learning models, The key parameter settings adopted for each method are listed in Table 2.

### 4.7. Hyperparameter Optimization

Furthermore, to evaluate the impact of hyperparameter optimization on the accuracy of parentage identification, we utilized GridSearchCV with five-fold cross-validation to perform hyperparameter optimization on the five machine learning methods using datasets generated with different training set sizes. Each method was trained using both default parameters and hyperparameter optimization under the same training set partition, and accuracy was evaluated on the same validation set. The hyperparameter search ranges are summarized in Table 3.

### 4.8. Accuracy Assessment

The accuracy of full-sib family assignment was evaluated separately for simulated datasets and real genotype data of sturgeons, following consistent evaluation criteria to ensure comparability across scenarios.

#### 4.8.1. Accuracy Assessment Using Simulated Data

For the simulated datasets, accuracy was assessed under different combinations of model parameters, including training set size (*nIND*), number of sires (*nSIRE*), number of dams (*nDAM*), and number of SNPs (*nSNP*). For each parameter combination, the entire simulation and analysis procedure was independently repeated 10 times. Each replicate consisted of data generation, dataset partitioning, model training, and prediction on the validation set. Prediction accuracy was defined as the proportion of individuals in the validation set whose full-sib family was correctly identified, calculated as:

$$\mathrm{Accuracy}=\frac{\mathrm{Number}\;\mathrm{of}\;\mathrm{correctly}\;\mathrm{assigned}\;\mathrm{individuals}\;\mathrm{in}\;\mathrm{the}\;\mathrm{validation}\;\mathrm{set}}{\mathrm{Total}\;\mathrm{number}\;\mathrm{of}\;\mathrm{individuals}\;\mathrm{in}\;\mathrm{the}\;\mathrm{validation}\;\mathrm{set}}$$

The final accuracy for each parameter combination was reported as the mean ± standard deviation (SD) of the validation accuracy across the 10 independent replicates.

#### 4.8.2. Accuracy Assessment Using Real Resequencing Data

For the genotype dataset of sturgeons, a family-based data partitioning strategy was adopted to evaluate model performance under realistic breeding conditions. Within each full-sib family, 10 individuals were randomly selected and fixed as the validation set. Training datasets were then constructed from the remaining individuals by randomly sampling *nIND* = 15, 10, 8, 6, 5, 4, and 3 individuals per family, respectively. For each training set size, model training and validation were repeated 10 times using independent random samplings of training individuals to account for sampling variability and ensure robust accuracy estimation. Prediction accuracy was evaluated using the same metric as defined above. The final accuracy for each training scenario was calculated as the mean validation accuracy across the 10 replicates.

## 5. Conclusions

This study demonstrates that machine learning approaches enable highly accurate full-sib family assignment using genome-wide SNP data, achieving identification accuracies exceeding 99.9% on validation datasets across multiple simulated scenarios. Our results indicate that training set size, SNP density, and family genetic structure are the dominant factors influencing assignment accuracy, whereas differences among algorithms and hyperparameter optimization play comparatively minor roles. Validation using real Russian sturgeon data further confirms the robustness and practical applicability of the proposed framework. These findings provide an effective genomic tool for pedigree reconstruction and family management in sturgeon breeding programs, which may facilitate inbreeding control, balanced family contributions, and the long-term maintenance of genetic diversity.

## Figures and Tables

**Table 1:** Number of individuals for each family.

Family	Number of Individuals
B1	32
B2	31
B3	29
B4	29
B5	31
B6	27
B7	35
B8	29
B9	29
B10	30
B11	30
B12	28
B13	30
B14	36
B15	30
B16	30
B17	30
B18	31
B19	35

**Table 2:** Parameters in different machine learning methods.

Method	Parameters
RF	n_estimators = 100, max_depth = None, random_state = 42
KNN	metric = ‘hamming’, n_neighbors = 5
XGBoost	n_estimators = 100, max_depth = 3, learning_rate = 0.1, use_label_encoder = False, eval_metric = ‘mlogloss’, verbosity = 0,
NB	default parameters
SVM	C = 1, kernel = ‘linear’

Note: RF: random forest; KNN: K-nearest neighbors; XGBoost: extreme gradient boosting; NB: naive bayes; SVM: support vector machine.

**Table 3:** Hyperparameter grid search settings for different machine learning methods.

Method	Parameters
RF	‘n_estimators’: range (10, 200, 10), ‘max_depth’: range (3, 21, 3), random_state = 42
KNN	‘n_neighbors’: (2, 3, 4, 5, 6, 7, 8, 9)
XGBoost	‘n_estimators’: (30, 50, 70, 85, 100, 120), ‘max_depth’: (3, 5, 7, 9), ‘learning_rate’: (0.05, 0.1, 0.2, 0.3, 0.5)
NB	‘var_smoothing’: np.logspace (−12, −6, 7)
SVM	C’: (0.1, 0.5, 1, 2, 5, 10), ’kernel’: [‘linear’, ‘rbf’], ’gamma’: [‘scale’, ‘auto’]

Note: RF: random forest; KNN: K-nearest neighbors; XGBoost: extreme gradient boosting; NB: naive bayes; SVM: support vector machine.

## Data Availability

The datasets used and/or analysed during the current study are available from the corresponding authors on reasonable request.

## Supplementary Materials

The following supporting information can be downloaded at: https://www.mdpi.com/article/10.3390/ijms27104359/s1.
